# Particle and Photon Detection: Counting and Energy Measurement

**DOI:** 10.3390/s16050688

**Published:** 2016-05-12

**Authors:** James Janesick, John Tower

**Affiliations:** 1 SRI-Sarnoff, 4952 Warner Avenue, Suite 300, Huntington Beach, CA 92649, USA; 2 SRI-Sarnoff, 201 Washington Road, Princeton, NJ 08540, USA; john.tower@sri.com

**Keywords:** ultra low noise, CCD, CMOS, imagers

## Abstract

Fundamental limits for photon counting and photon energy measurement are reviewed for CCD and CMOS imagers. The challenges to extend photon counting into the visible/nIR wavelengths and achieve energy measurement in the UV with specific read noise requirements are discussed. Pixel flicker and random telegraph noise sources are highlighted along with various methods used in reducing their contribution on the sensor’s read noise floor. Practical requirements for quantum efficiency, charge collection efficiency, and charge transfer efficiency that interfere with photon counting performance are discussed. Lastly we will review current efforts in reducing flicker noise head-on, in hopes to drive read noise substantially below 1 carrier rms.

## 1. Introduction

Silicon CCD and CMOS imagers have been demonstrated to be exceptional detectors for particle counting and energy measurement for some time. The spectral range where photon counting is possible covers an extensive wavelength range from 0.1 to 1000 nm (1.24 to 12,400 eV), *i.e.*, nIR, visible, UV, EUV and soft X-ray. At the beginning of the EUV range (10 eV) photon energy absorbed by the imager can be determined by using the simple relation [1],

(1) $$E(\text{eV})=3.65n_{i}$$

where 3.65 is an experimentally determined constant for silicon (eV/carriers) and *n_i_* is measured quantum yield (carriers generated/interacting photon). The equation is applicable to photon energies greater than ~10 eV. The formula is not useful for energies less than this because the constant 3.65 wildly fluctuates. Besides photons, this equation is also useful for any particle that ionizes silicon atoms (electrons, protons, muons, *etc.*). The uncertainty in energy measurement is limited by the detector’s read floor and Fano noise. Fano noise, the variation of charge generated per photon, is found by,

(2) $$FN={\left(Fn_{i}\right)}^{0.5}$$

where *F* is the Fano factor (~0.1 for silicon) and *n_i_* is the quantum yield (carriers generated per photon). Physically Fano noise arises within the silicon where a small amount of thermal energy is lost to the silicon lattice (phonons) instead of creating electron-hole pairs. The variation in the loss from pixel to pixel is the Fano noise generated and represents a fundamental noise source in determining the energy of high energy particles [2].

**Figure 1:**
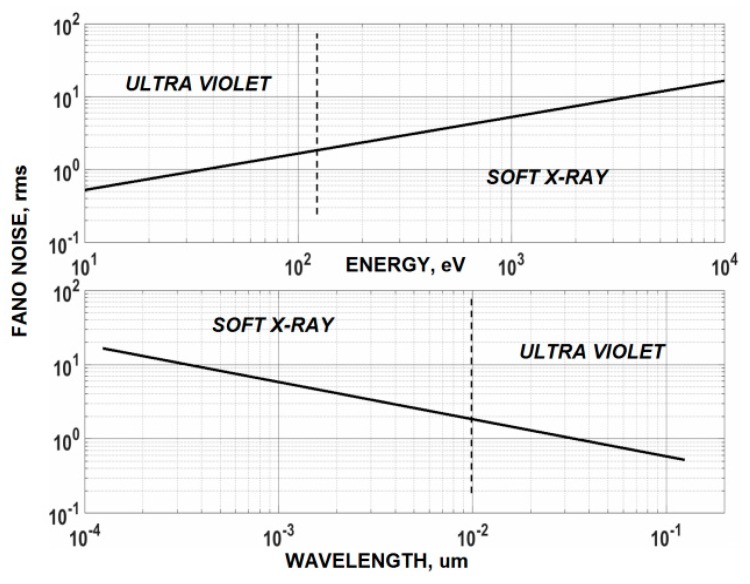
The plots above are used to determine the “Fano noise limit” for an imager with a given read noise. For example, a read noise of one carrier rms will cover the soft X-ray and extend into the UV at wavelength of 0.03 um (~40 eV).

**Figure 2:**
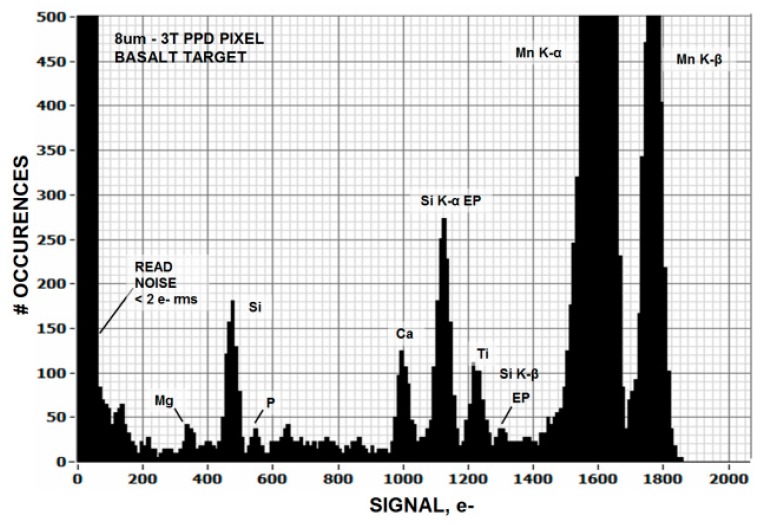
Photon counting and energy histogram generated by a 3T PPD CMOS pixel imager demonstrating Fano-noise limited performance over the entire soft X-ray regime.

Imagers where Fano noise is greater than the sensor’s read noise are referred to as “Fano noise limited” [2]. Figure 1 plots Fano noise as a function of photon energy (eV) and wavelength (µm) showing the Fano limited range that can be covered by an imager for a given read noise floor. For example, Figure 2 presents a histogram taken from a Fano noise limited 8 um 3T NMOS pinned photo diode (PPD) pixel array showing multiple energy lines from a basalt target fluoresced with 5.9 keV Mn X-rays. The sensor’s read noise is slightly less than 2 electrons (e^−^) allowing Fano limited performance to cover the entire soft X-ray range (0.12 nm–10 nm). This spectral range has been particularly fruitful for CCD soft X-ray imaging spectrometers used in scientific and space applications. The width of each spectral line revealed in Figure 2 is a measurement of the amount of Fano noise generated. The spectral range for this imager can be further extended into the EUV range (10 nm–124 nm) if only photon counting is desirable.

The photon energy for visible (400 nm–700 nm) and nIR (700 nm–1100 nm) wavelengths is only able to generate one electron-hole pair/photon, limiting sensing to only photon counting. But when leaving the visible range into the UV multiple carriers per photon are generated allowing their energy to be determined. The challenge left today is to extend energy measurement into the UV and provide photon counting in the visible/nIR wavelengths by reducing the sensor’s read noise floor.

**Figure 3:**
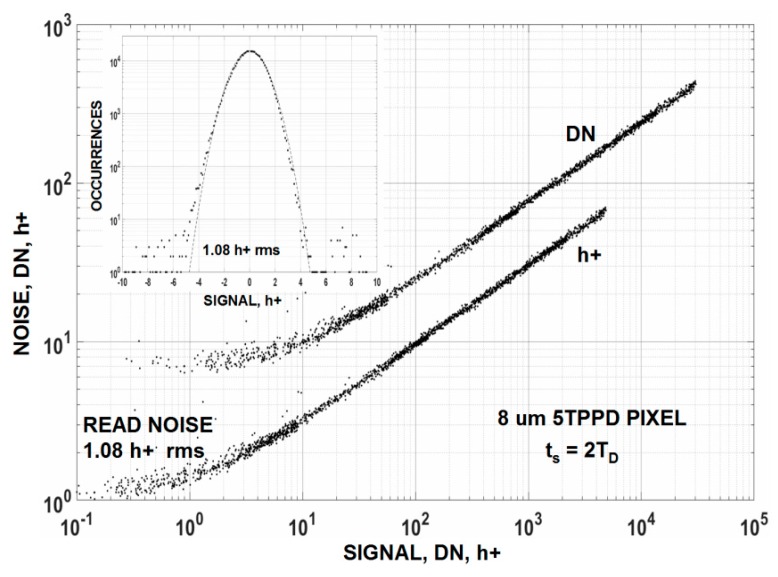
Photon Transfer curves demonstrating a read noise floor of ~1 hole rms. Most high performance CCD and CMOS imagers are close to this noise level.

The average noise floor for high performance CCD and CMOS imagers is typically shy of achieving “one” carrier of noise (this excludes EMCCD and SPAD detectors). For example, Figure 3 presents a photon transfer (PT) [2,3] curve generated by a 512 um × 512 um × 8 um PPD PMOS CMOS imager with a read noise floor of 1.08 holes (h+ )at room temperature. It is extraordinary that the CCD/CMOS imaging community for the most part has achieved approximately “one” carrier of noise considering the multitude of solid state phenomena at play at world wide fabrication foundries for several years (decades in the case of CCDs). But why “one”? Is this apparent final outcome simply coincidental? One also wonders further why “one” carrier of noise along with “one” carrier of signal forces the minimum detection limit (MDL) of the detector to be “one” (*i.e.*, S/N = “one”). The conundrum continues with why today’s imagers are very close but yet so far from “routinely” counting single visible photons consistently across full imaging arrays. 

**Figure 4:**
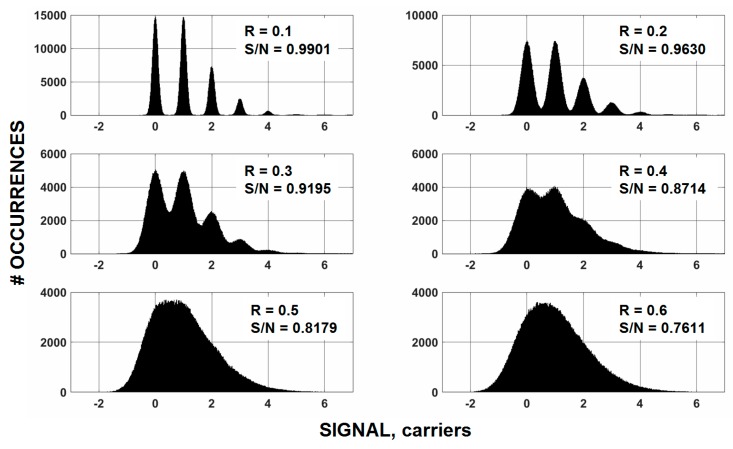
Simulated histograms for an average signal level of “one” carrier and six different read noise (R) levels. The histogram shows that a read noise <0.3 carriers rms is required for precise photon counting results.

To appreciate the challenge, Figure 4 displays computer simulated histograms for different read noise levels for an average signal level of “one” interacting photon/pixel and quantum yield of “one”. These plots show that it is necessary to have a read noise floor of <0.3 e^−^ before the histograms appear “quantized”, which in turn sets the stage for visible/nIR photon counting. Also notice from these plots that the net signal-to-noise (S/N) determined directly from the simulated plots hardly changes as read noise is lowered from 0.3 to 0.1 carriers because the presence of photon shot noise. Nonetheless, read noise improvement in this range is especially critical to future visible/nIR photon counting imagers and applications. 

**Figure 5:**
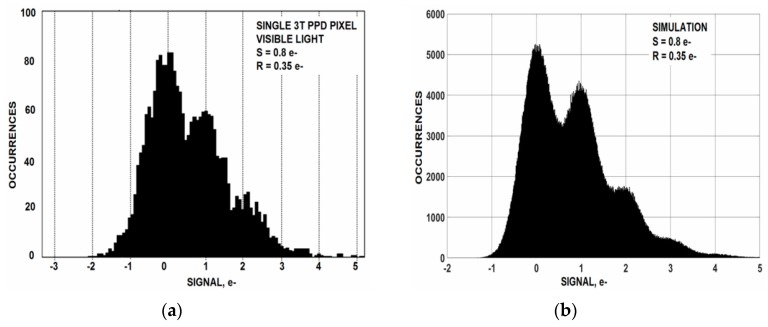
Experimental (**a**) and simulated (**b**) histograms for a low noise 3T PPD CMOS pixel showing a Poisson distribution profile. Read noise for this pixel is 0.35 e^−^ rms.

**Figure 6:**
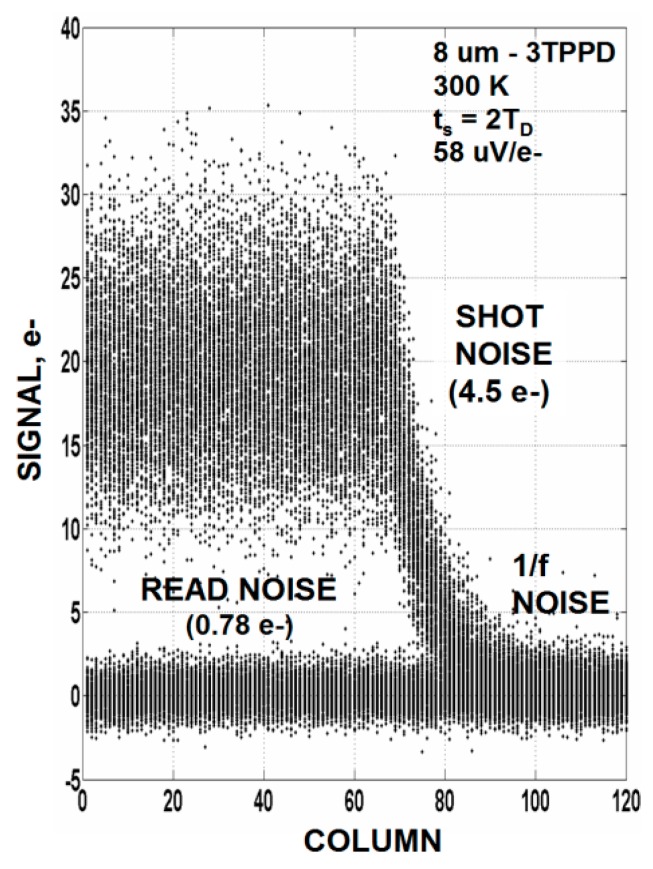
Raw video response for a sub carrier noise 3T PPD pixel in response to a light source that slowly turns off. Read noise for this pixel is 0.78 e^−^ rms.

Occasionally we do run into an individual low noise pixel that is capable (but barely) of photon counting allowing us to see the Poisson distribution profile that governs photon counting statistics. The top histogram of Figure 5a is generated by an exceptionally low noise “cherry picked” NMOS 3T PPD pixel with a 0.35 e^−^ noise floor taken under cooled conditions (~−80 °C). An average signal level of 0.8 e^−^ is adjusted for the experiment (*i.e.*, 0.8 interacting photons/pixel). The lower histogram shown in Figure 5b is a computer simulation showing that data and theory match up quite well. Figure 6 is a different NMOS 3T pixel illustrating how 4.5 e^−^ photon shot noise folds into the 0.78 e^−^ noise floor as the light source slowly turns off. These low noise pixels imply that the “one” carrier noise level that manufacturers have produced may not represent a fundamental limit but simply related to the noise level being “good enough”. This stance is reasonable to assume because for normal imagery the S/N is always less than “one” regardless of how much lower the read noise is beyond “one”. Hence, imager manufactures may not feel obligated to aggressively lower read noise any further (especially monetarily). This position produces an illusion that there may be a physical barrier of some kind holding us to “one” noise carrier. Fairly recently (2015) it has become more apparent that the “one” noise barrier has been a deception for groups are now reporting sub carrier performance down to 0.23 e^−^ for selected pixels [4,5] allowing for visible photon counting. The future objective for sensors with sub carrier read noise floors is to have all pixels contained on an imager exhibit close to the same low noise level and not just a few while at the same time achieve a reasonable saturation level for high dynamic range.

## 2. Read Noise Reduction

### 2.1. Flicker and Random Telegraph Noise

**Figure 7:**
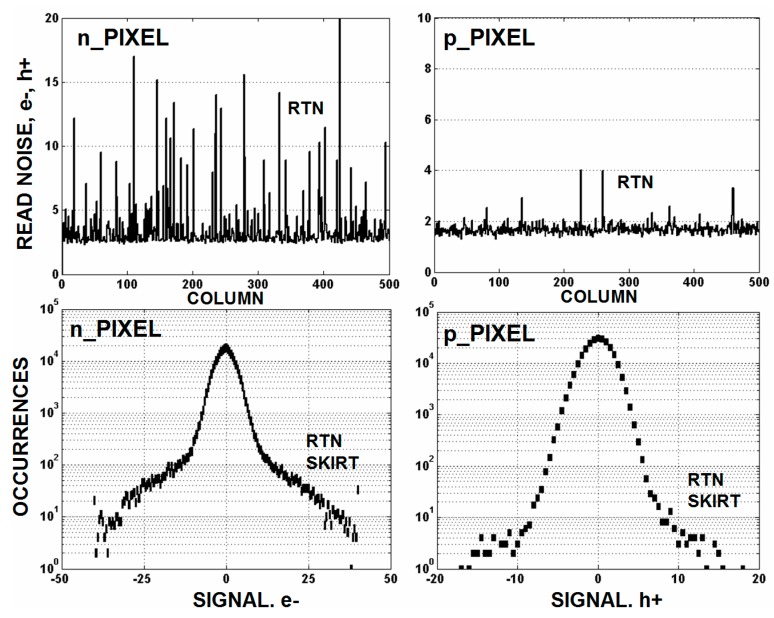
Random telegraph noise (RTN) population comparison for NMOS (n_pixel) and PMOS (p_pixel) pixels.

Flicker and random telegraph noise (RTN) generated by the pixel’s source follower (SF) MOSFET are the dominant noise sources that limit read noise performance for an imager. It is generally believed that RTN is associated with traps within the gate oxide. Free carriers from the SF channel tunnel in and out of these traps at different rates depending on the tunneling distance to the trap and the barrier height encountered. The local “on” and “off” potential in the localized region of a trapped carrier modulates the SF channel current in a digital manner and is seen as RTN output voltage fluctuation (with varying amplitude and frequency from pixel to pixel depending on the trap time constant and location). NMOS imagers have a great deal more RTN pixels than PMOS devices do. For instance, Figure 7 compares the read noise for NMOS and PMOS pixels showing very few RTN pixels for the PMOS imager. The background flicker noise levels are different for the plots because the sense node (SN) conversion gains are not the same. However, it is observed that when the sensitivity is the same the background flicker noise level (rms carriers) is nearly identical for PMOS and NMOS pixels.

Other sources of pixel noise have been observed that mimic SF RTN. For example, a poor SN metal contact exhibits RTN behavior. Leakage currents related to the pixels transfer gate (TG) and reset gate that are in close proximity to the SN can also contribute noise for some local pixels. However, one can usually differentiate these noise sources from SF noise by varying the voltages to these gates and the SN reset bias level and noting the affect.

The reason for the NMOS/PMOS RTN population difference is holes are much less likely to tunnel into oxide traps than electrons because the barrier height is greater. On these grounds we do not need to consider RTN as a fundamental barrier to lowering read noise. Therefore, the problem of beating down SF flicker noise has become the only fundamental noise source in reducing the read noise floor for PMOS imagers. However, for NMOS devices we must use other strategies to reduce RTN. For example buried channel MOSFETs can be employed which to some degree isolates the SF current carrying channel from the surface. In turn this increases the barrier to the traps and lowers RTN. We will briefly discuss this technology below. 

Two primary theories describe flicker noise generation in MOSFETs: Hooge’s mobility fluctuation and McWhorter’s carrier density (or number) fluctuation models [6,7]. The latter mechanism is based on the random trapping and release of conduction band carriers located at the Si-SiO_2_ interface of the SF MOSFET (*i.e.*, generation recombination (GR) noise). The former is related to conductance or resistance fluctuations within the channel of the SF MOSFET. There are several possible causes for channel mobility noise. For example, fluctuations may be associated with a lower surface mobility compared to bulk silicon because carriers scatter off the Si-SiO_2_ interface. Another possibility could be linked to the SF channel’s implanted impurities that are randomly imbedded in the silicon lattice. The depleted ions in the SF channel produce small individual potential barriers that “fluctuate” current flow thus producing noise [8].

**Figure 8:**
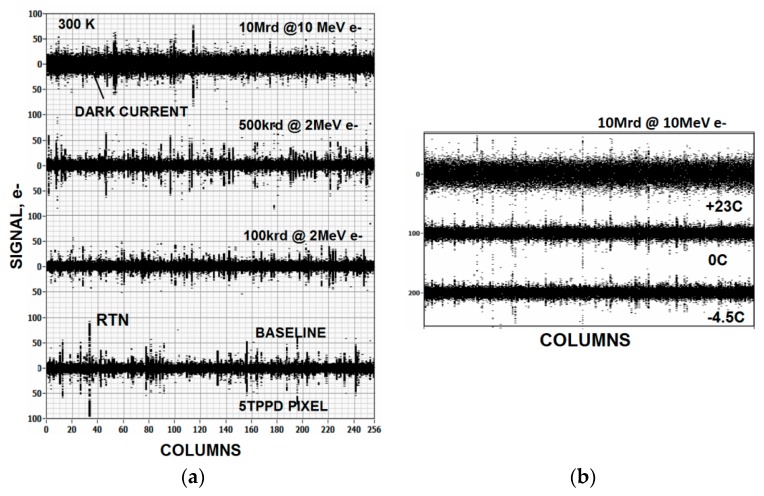
High energy electron damage source follower (SF) noise responses taken from a CMOS 5TPPD image. As the radiation dose level dramatically increases there is no significant increase in read noise even though gate oxide damage is taking place. The lower plot of (**b**), which is taken at −4.5 °C, shows that read noise at 23 °C for the 10 Mrd exposure is limited by dark current noise. Cooling the detector eliminates this dark current noise source resulting in the SF read noise level.

To help identify where 1/*f* noise sources are located, pixels have been irradiated with high energy radiation sources to deliberately increase interfacial Si-SiO_2_ surface states. In general when such experiments are performed we find that the read noise level does not change. For example, Figure 8a shows that the read noise remains constant after irradiating an unbiased NMOS CMOS imager to different dose levels of high energy electrons. The noise data shown was generated using a very short exposure time (~10 µs) such that the increase in dark current shot noise due to radiation damage was negligible. However, the read noise for 10 Mrd exposure appears to be higher because thermally generated dark current was high enough to become an issue. Cooling the pixels slightly as displayed in Figure 8b lowers the noise down to the same level as other irradiated imagers. 

The same sensors irradiated did exhibit charge transfer efficiency (CTE) degradation related to the TG demonstrating that surface states were in fact being generated. Some flat-band shift due to positive fixed charge buildup in the oxide induced by the radiation was also measured again indicating some damage took place but without a read noise increase. Other research groups have experienced the same surprising outcome [6]. Although far from being proven these findings begin to bias us to believe that the background flicker noise may not be dominantly associated with the oxide but instead within the current carrying channel of the SF. 

**Figure 9:**
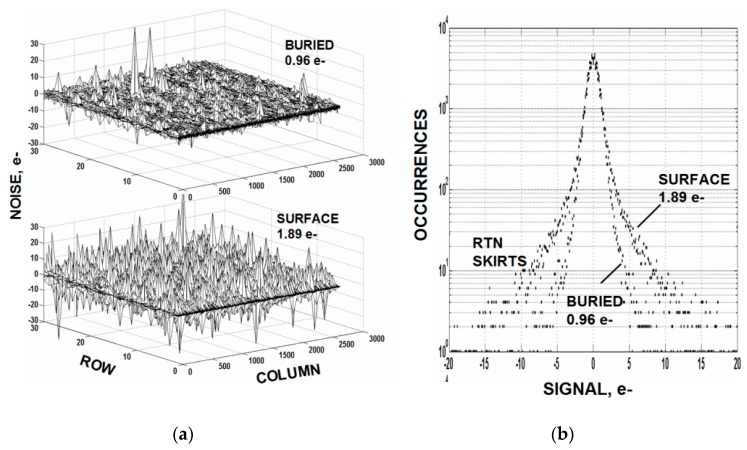
Noise performance comparison for surface and buried channel SF MOSFETs. The data shows that the buried channel device has reduced RTN but not reduced 1/*f* noise.

**Figure 10:**
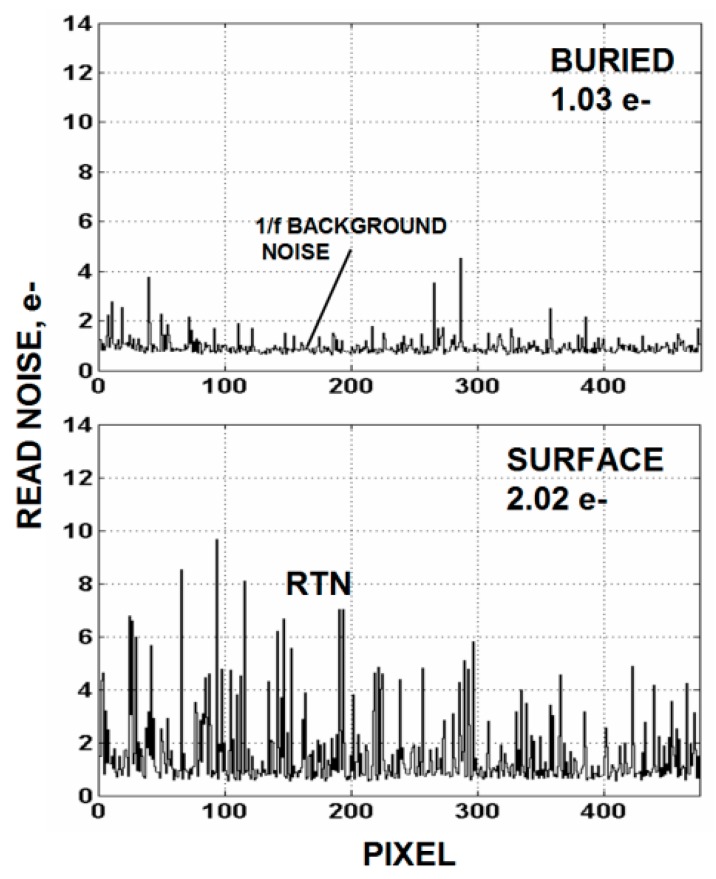
Buried (**top**) and surface (**bottom**) channel SF data showing more clearly that 1/*f* background noise is unaffected by buried channel use.

We have had some past success in reducing read noise using buried channel NMOS MOSFETs. Others working with this approach have realized the same benefit [9]. For example, Figure 9a,b shows the temporal noise difference for surface and buried channel MOSFETs. Notice that a significant RTN noise reduction takes place with buried use but does not change the background 1/*f* noise. Figure 10 also shows an unchanged background noise level for a row of 500 pixels where the rms noise level is determined for each pixel. The outcome is reasonable for RTN because the barrier increase provided by buried channel should diminish electron tunneling into the oxide. But why not a 1/*f* noise reduction? Possibly the buried channel employed here did not provide a sufficient barrier or shield deep enough for bulk generated 1/*f* noise. Or perhaps the 1/*f* noise is being generated in another location or mechanism other than just traps.

### 2.2. Correlated Double Sampling

**Figure 11:**
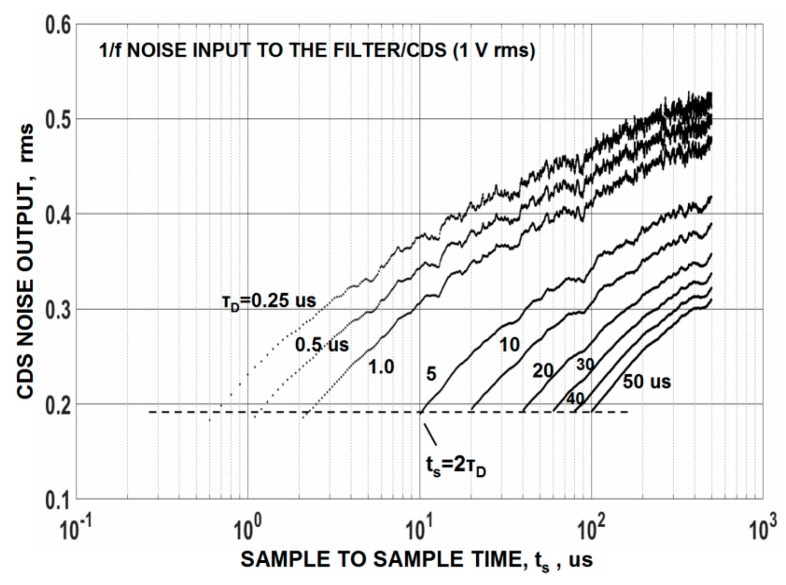
Computer simulated correlated double sampling (CDS) output noise in response of 1/*f* noise [9]. The lack of correlation on 1/*f* noise between samples is the cause for increase.

Flicker noise and correlated double sampling (CDS) share a fascinating relationship. Analysis and measurements show that optimum flicker noise reduction takes place when:

t_s_ = 1/(8B) (3)

where t_s_ is the time difference between samples before and after charge is transferred to the SN and B is the net equivalent noise bandwidth for the sensor and CDS system [10]. Equation (3) can also be expressed as t_s_ = 2τ_D_ where τ_D_ is the system dominate time constant. Figure 11 plots CDS output noise as a function of t_s_ for various τ_D_ for a flicker noise input. For a specified τ_D_ the output noise increases with t_s_ because 1/*f* noise correlation is being lost between the two samples. For very long t_s_ time the CDS output noise will eventually level off to 2^0.5^ times the input noise because the samples are fully uncorrelated and differenced. It is important to point out from this plot that CDS output noise is constant with t_s_ given that t_s_ = 2τ_D_ is fixed (*i.e.*, the dotted horizontal line shown on the plot). Under this timing boundary condition 1/*f* noise decreases with τ_D_ at the same rate as 1/*f* noise increases with t_s_. This is the central reason why it’s not feasible to eliminate 1/*f* noise by CDS processing. It should be mentioned that sampling less than 2τ_D_ results in a signal gain loss and lower S/N performance (the reason why the plots are bounded by the dotted horizontal line). Also, sampling an exponentially changing video waveform can produce camera instabilities (e.g., offset control).

**Figure 12:**
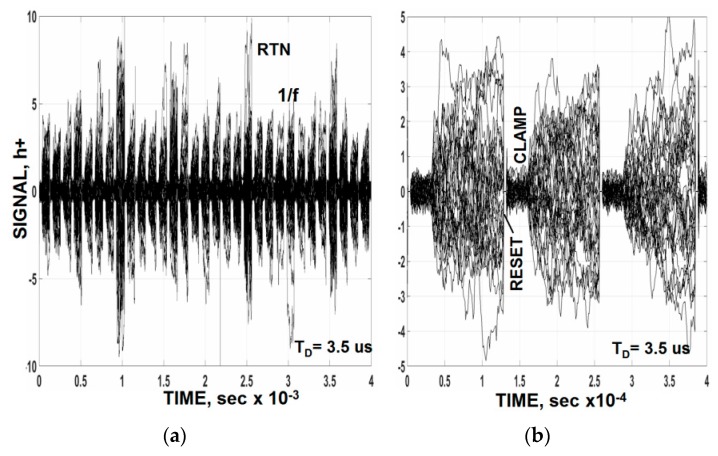
Raw video showing how noise increases after clamp is released for several pixels. (**a**) stacked raw clamped dark video plots; (**b**) magnified plot.

**Figure 13:**
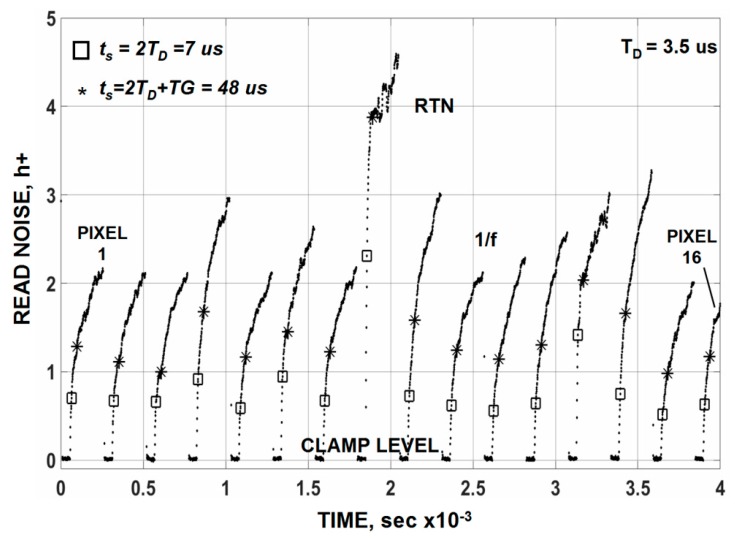
Measured noise for the raw clamped video in Figure 12. The “square” points represent the ideal sampling time after clamp is released (*i.e.*, t_s_ = 2τ_D_). The “star” points include TG overhead and settling time.

To demonstrate the claim above, stacked raw clamped dark video plots for a number of PMOS pixels is presented in Figure 12a. The response shown is how the noise would appear on a high persistence oscilloscope before it enters an analog-to-digital converter with internal sample and hold. The magnified plot presented in Figure 12b shows that timing starts by resetting and clamping the pixels’ video. The white noise seen in the clamp period is associated with the clamp switch (~0.1 h+). As one would expect 1/*f* noise slowly increases after the clamp release due to lack of correlation. Figure 13 plots this read noise buildup for sixteen of the pixels. Ideally the video should be sampled as soon as the clamp is released after the required video τ_D_ settling time is satisfied for lowest noise performance (*i.e.*, t_s_ = 2τ_D_ = 7 µs in this case). This special timing condition is illustrated by the “squares” on the plot. An average noise level of ~0.9 h+ is measured for a row of 500 pixels of which the sixteen pixels are contained. The data points with “stars” include the pixel’s TG overhead time where a higher noise is measured (1.7 h+) because the 1/*f* noise is less correlated. It should be also mentioned that averaging multiple samples in Figure 13 does not help to reduce the read noise floor. This is because the samples are semi correlated and the noise within the sample increases for each new sample taken. As a result the noise increases with sample number at the same rate as the noise reduction offered by averaging (*i.e.*, N^1/2^). It is very difficult to circumvent flicker noise presence for any signal processing scheme employed.

It is interesting to point out that using a 3T pixel for some applications can achieve a slightly lower noise than a 4T pixel simply because the 3T does not need to contend with TG overhead settling time. This assumes that the pixels are individually read out one by one by undergoing the entire exposure and CDS timing process (reset, clamp, flash expose and sample) before moving onto the next pixel. This approach is often used to test CMOS imagers including charge transfer pixels. Many figures in this paper are generated this manner to avoid dark current problems at room temperature.

**Figure 14:**
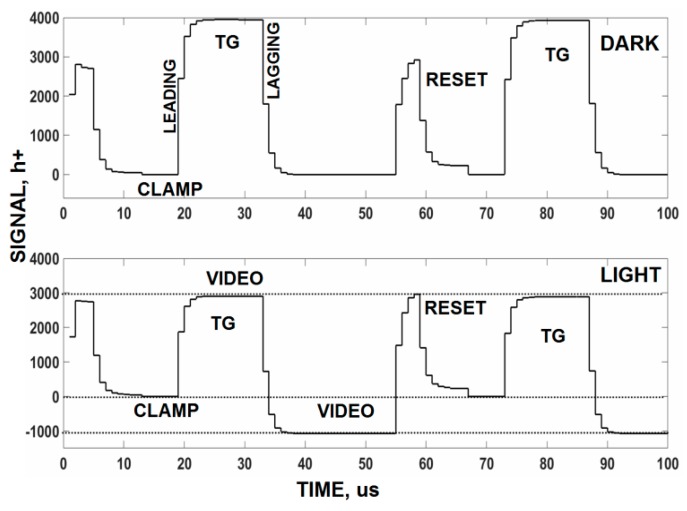
Dark (**top**) and light (**bottom**) raw video responses showing the pixel’s clock feedthroughs and the video level.

**Figure 15:**
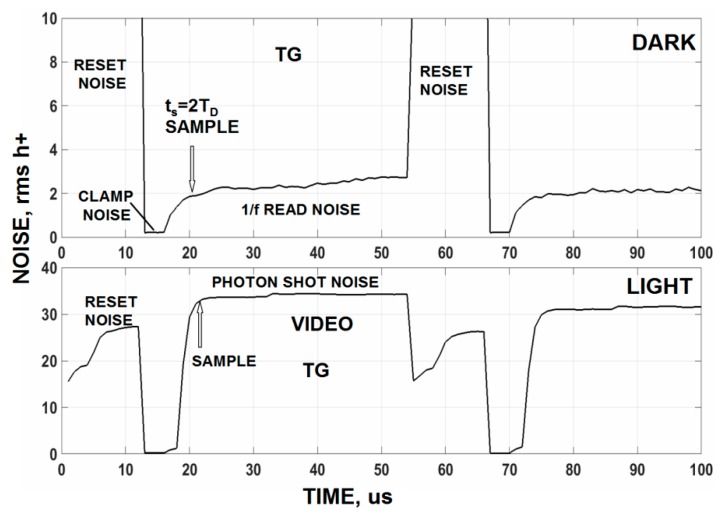
Noise levels for signal shown in Figure 14. The top dark plot shows how the read noise increases throughout the TG time period. No excess TG clock feed-through noise is observed.

Although not conventional, one can shorten the TG overhead time by sampling the video on the leading edge of the TG instead of the lagging edge. In fact sampling can be performed at any time throughout the TG clock period as long as the t_s_ = 2τ_D_ settling time is satisfied. Figure 14 presents raw video traces taken under dark and light conditions. The traces show the reset clock feedthrough, clamp period, TG clock feedthrough and the downward change in video level to the light level applied. Figure 15 is the corresponding noise measured for Figure 14. Note after the clamp is released that the read noise increases due to correlation loss. The presence of a large TG clock feedthrough does not influence the noise measured because it only represents a changing offset which can be readily removed by a computer.

### 2.3. Conversion Gain

The conversion gain (V/carrier) depends on the various parasitic capacitances attached to the pixel’s floating diffusion SN besides the SF gate capacitance. It is our experience that the highest conversion gain realized does not bear the lowest average noise as long as the SF gate capacitance dominates. Instead an optimum SF size exists for lowest noise. Also in general, 1/*f* and SN sensitivity scale together for a large range of SF width and lengths. This strong relationship between 1/*f* and V/carrier has clearly been experienced when CCD and CMOS read noise performance is compared. The former technology typically has a considerably lower conversion gain than the latter yet both technologies in essence are limited to the same read noise floor (*i.e.*, ~1 carrier rms). This outcome is because flicker noise is proportional to SF gate capacitance while the SN conversion gain is inversely proportional to it. SF MOSFET size and gate capacitance have grown smaller as imager technology improves but without a significant reduction in read noise.

### 2.4. Nondestructive Readout

**Figure 16:**
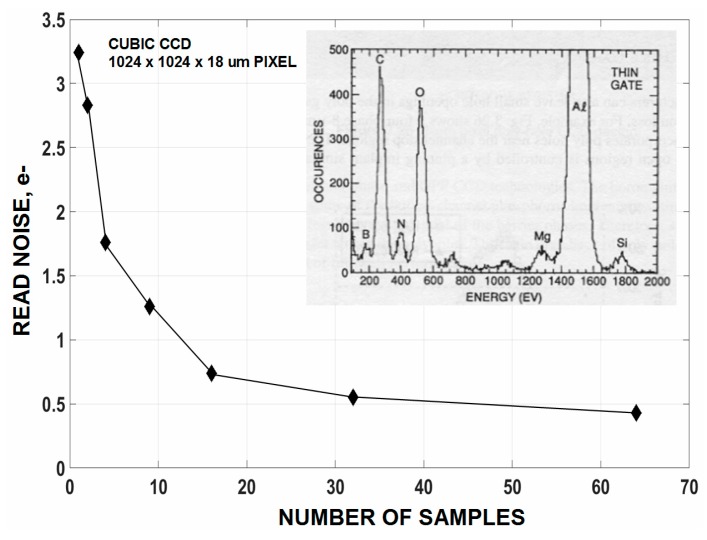
Nondestructive readout of the CUBIC imager showing how the read noise decreases with the number of samples averaged [1]. Embedded in the figure is a low energy soft X-ray histogram showing B, C, N and O lines with Fano-noise limited performance.

Nondestructive floating gate readout schemes have worked in a straightforward fashion in producing sub carrier noise performance assuming there are no restrictions involving frame time requirements and operating temperature. The technology coined floating gate “Skipper” long ago can theoretical achieve any desired noise level as the read noise decreases by the square-root of the number of samples taken for each pixel [2]. Figure 16 presents the read noise for a Skipper CCD where ~0.4 e^−^ is achieved with 64 samples beginning with a 3.25 e^−^ noise floor for a single sample [1]. Unfortunately no attempt was made to count single visible photons with this imager since it was busy counting X-ray photons for a soft X-ray flight mission called Cosmic Unresolved Background Instrument (CUBIC). The SN conversion factor is quite low for the device (3 uV/ e^−^) because of large parasitic SN and SF gate capacitances. More recent Skipper CMOS pixels have shown that the conversion gain for a floating gate and a floating diffusion are about the same. Hence, it may be possible for a 1 carrier noise Skipper device to achieve 0.3 e^−^ with nine samples using today’s fabrication technology without reducing 1/*f* noise. These Skipper CMOS pixels were fabricated using a multi transfer buried channel gate process developed with Jazz Semiconductor for CMOSCCDs. No serious attempt has been made to characterize these pixels for photon counting use as of yet (only single sample data has been taken). The Skipper noise reduction approach was employed in the far past when the SN conversion was very low as sub micron fab technology did not exist. Today one can achieve the same 0.3 e^−^ noise floor with one sample with much higher conversion gain [5].

## 3. Additional Counting Issues

Besides read noise, quantum efficiency (QE), CTE and charge collection efficiency (CCE) interfere with photon counting precision. We will take a look at these problems in this section. 

### 3.1. Quantum Efficiency

QE is never ideal and hence sensors will fall short in precisely counting the number of incoming photons. Photons can be reflected, absorbed by non-active regions of the pixel or entirely transmitted through the active silicon in lowering QE. QE is dependent on silicon wafer thickness, photon wavelength and the pixel technology utilized (e.g., backside *versus* frontside illumination). Fortunately a photon miscount due to QE is probably not that critical to most future applications given that today’s imagers exhibit fairly high QE performance. 

### 3.2. Charge Transfer Efficiency

CTE is rarely perfect either (3T CMOS pixels are not vulnerable to CTE problems since charge is not transferred). A CTE issue usually translates to deferred carriers rather than their absolute disappearance. Seldom do signal carriers recombine but are instead trapped and released at later time. For CCDs trapped charge is spatially and time deferred whereas carriers are only time deferred for CMOS pixels. Also CCD CTE degrades as signal level decreases whereas CMOS CTE typically increases. Lastly, CMOS usually involves only a single transfer within the pixel where CCDs must transfer charge hundreds or thousands of times in taking charge to the output amplifier which only exasperates other CTE issues. Also the number of transfers is especially critical when a sensor operates in a damaging high energy radiation environment. For these reasons CMOS imagers are highly favored over CCD for single photon detection work. 

**Figure 17:**
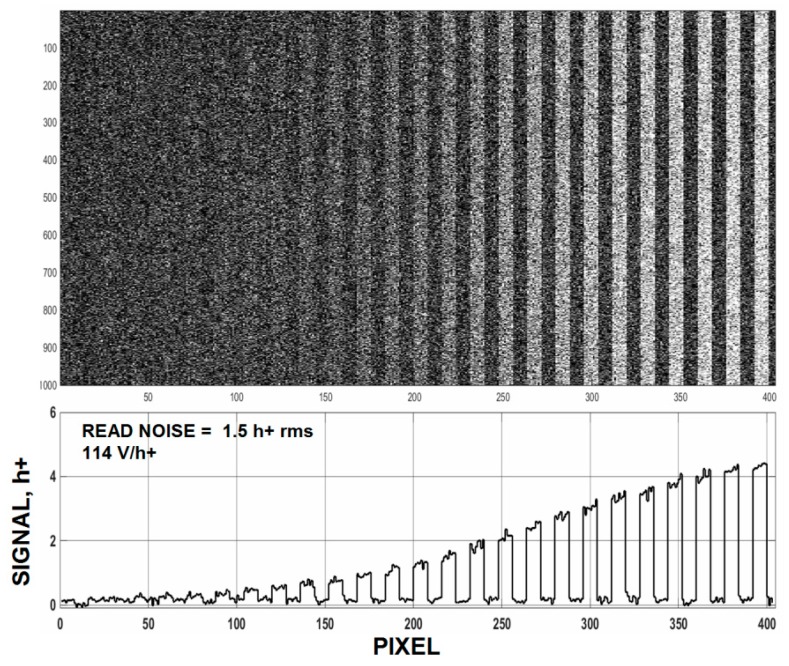
Low light image for a 10 um 5T pinned photo diode (PPD) PMOS pixel imager.

Nonetheless, a few CMOS CTE issues still can hamper photon counting today. For example, the pixel’s TG often has a front edge built-in thermal potential barrier that slows charge carrier transit time to the SN region. The barrier height observed is a complex function of PPD and TG implants (dose, energy and alignment), silicon resistively, specific details with the layout of the pixel and the fabrication process employed. Even so, trial and error design and processing can sufficiently reduce the barrier to an acceptable level for a given application. Also unlike buried channel CCDs the TG usually runs surface channel. That is, charge is transferred at the Si-SiO_2_ surface where traps abound. However, if charge transfers very quickly through the TG the probability that a carrier meeting up with trap can be kept very low yielding imagers with immeasurable CTE for small charge packets (our measurement limit is CTE ~0.999). It is more demanding when large charge packets are transferred because CTE degrades with signal as collapsing fields between the photo diode and SN slows transit time increasing the chances of trap interaction. Few sensors have been tested where CTE is next to perfect as full well is approached. Figure 17 demonstrates a high performance 10 um PMOS pixel imager without a low light deferred CTE problem. Notice as the light level decreases across the chip the signal slowly disappears into the read noise floor (1.5 h+ for this imager).

Either inverting a buried channel or accumulating surface channel poly gate with free carriers from the substrate has been important clocking scheme to achieving low dark current generation for both CCD and CMOS technologies [10]. For our PMOS pixels accumulating a surface channel TG takes place at 4.7 V for a *p*_doped TG and 3.7 V for *n*_doped TG assuming a substrate voltage of 3.3 V. However, doing so promotes an inconspicuous but adverse CTE problem that is particularly important to night vision and future photon counting applications. For the case of a PMOS pixel, accumulation (ACC) takes place when electrons from the substrate diffuse and collect within TG surface state traps. This in turn arrests TG dark current. Also any presence of trapped holes in the gate oxide in the form of image lag (deferred charge) is completely annihilated by the presence of these electrons. However, this action can deceive the user thinking that perfect CTE without image lag is being achieved when in fact a loss of charge is taking place. When the TG is clocked low to transfer signal charge the majority of these electrons return back to the substrate. However, some electrons remain trapped which can now recombine with the signal holes being transferred at the same time. It is difficult to determine the amount of absolute loss that is taking place as it is similar to experiencing a QE loss at very low light. The percentage of charge that is lost typically increases with decreasing signal and can be significant without the user even realizing it (tens of percent have been experienced). Low energy X-rays can be used for an acid test to determine absolute CTE loss. But a rather easier method to find loss in a relative sense is by measuring the signal transferred as the TG barrier voltage is varied. 

**Figure 18:**
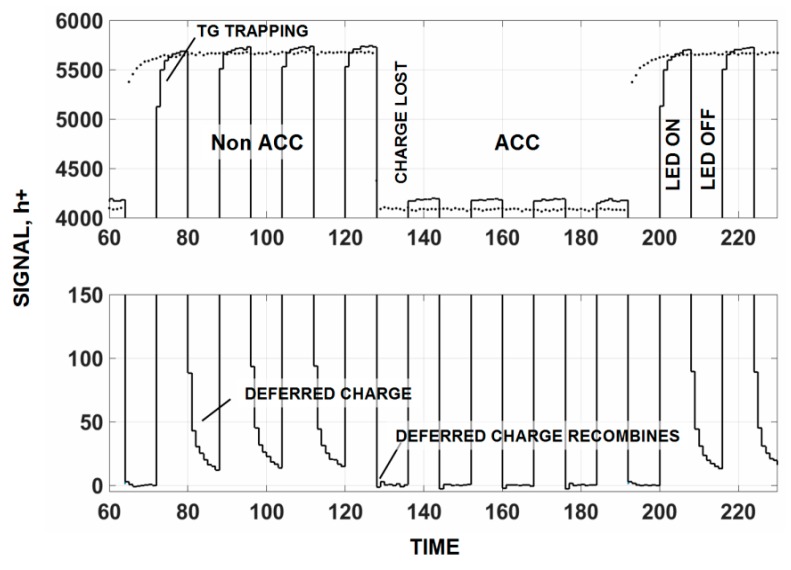
Response for a troubled imager when the transfer gate (TG) is switched in and out of accumulation. Deferred and recombination loss are clearly seen for the device. The top trace shows how charge is trapped whereas the lower trace shows how trapped charge is released and deferred in time.

For example, Figure 18 presents a response for single CMOS 4T pixel as the light to it is turned on and off as indicated. TG not accumulated (non ACC) and accumulated (ACC) responses are presented as the pixel is switched between these two modes. This particular pixel has a CTE problem associated with the TG front edge barrier discussed above and is seen in Figure 18 as deferred charge tails after the light is turned off for the non ACC mode. Charge loss is observed when TG is biased into ACC as trapped electrons recombine with signal holes. Also the deferred charge tail disappears as electrons recombine with trapped holes. 

**Figure 19:**
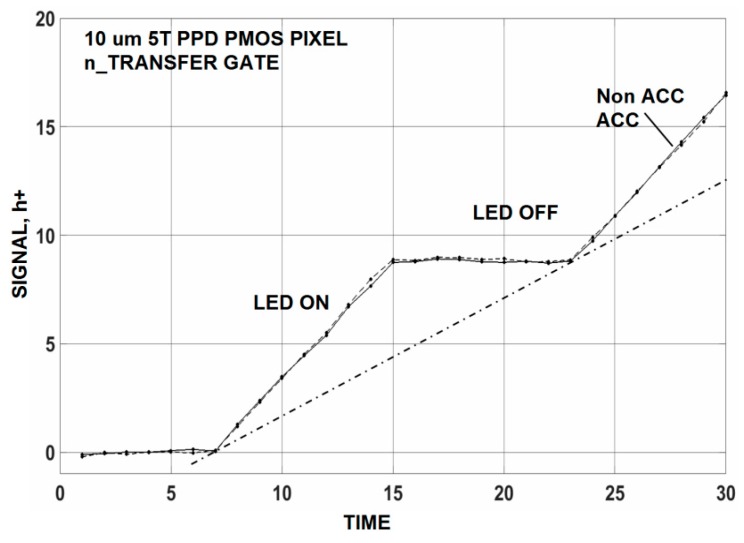
Ultra-low light accumulation (ACC) and non ACC responses without recombination loss.

**Figure 20:**
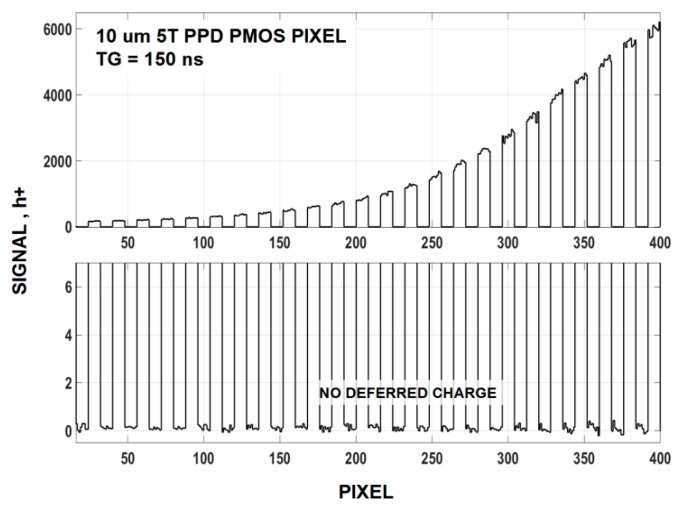
Response from the same imager as Figure 19 showing no deferred charge up to 6000 h+. Charge transfer efficiency (CTE) performance loss for this imager is immeasurable.

In general, the amount of recombination loss and deferred charge increase as TG charge transfer transit time increases. We find that a transit time of ~100 ns is sufficient to avert these problems. For example, Figure 19 compares ACC and non ACC settings for a single 10 um PMOS pixel that is transferring single carriers to the SN on the average. The SN is not reset for this test allowing charge to build on the SN in a linear fashion as shown. The light source (LED) is turned “on” and “off” as SN integration takes place on the SN. We can safely conclude that no recombination is taking place when the ACC and non ACC curves are compared. Figure 20 is taken from the same imager biased in the non ACC mode and exposed and stimulated with a high light level source. There is no deferred charge (*i.e.*, lag) detected for the imager and yield a CTE >0.999 over its dynamic range (full well is 15,000 h+). This imager would be ideally suited for photon counting if the read noise was lower (1.4 h+ read noise is measured). 

### 3.3. Charge Collection Efficiency

**Figure 21:**
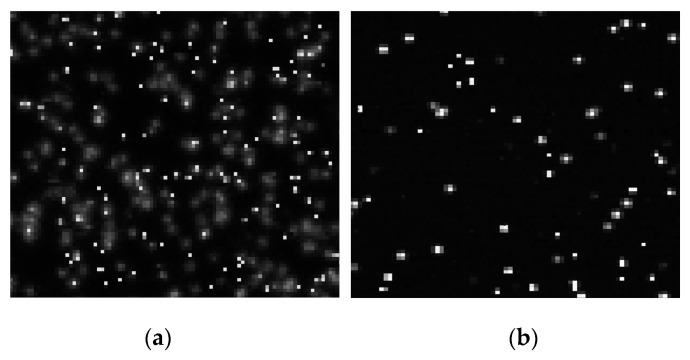
Mn (Fe-55) soft X-ray responses taken from an 8 um 3TPPD pixel image fabricated on 25 and 15 µm epitaxial silicon. Charge collection efficiency (CCE) performance for the thicker imager (**a**) is inferior compared to the thinner device (**b**).

CCE is by no means perfect. Ideally carriers generated by a photon should only be collected by the target pixel without sharing with its neighbors. MTF, OTF, point spread and edge response measure CCE performance and signify the degree of sharing that takes place. Pixel size, silicon thickness/resistivity and the potential difference across the active silicon all play a role on CCE. A CCE problem is especially influential on high energy photon detection applications since a charge packet can diffuse and divide with neighboring pixels (referred to as “split” events). For instance, Figure 21a shows 5.9 keV Mn X-ray events generated by an 8 um 3T PPD pixel fabricated on 25 um intrinsic epi silicon. One can see many split events where the 1620 e^−^ Mn charge packets diffuse to other pixels instead of being fully contained in the target pixel. Figure 21b presents a response when the same pixel is fabricated on 15 um intrinsic epi silicon where CCE performance is much improved. Neighboring pixels must be summed with the target pixel to determine the energy of the photon. However, this off-chip summation results in an increase in read noise (by the square-root of the number pixels summed) and a corresponding loss of energy resolution. Also split events may experience some recombination loss which leads to an absolute error in energy measurement. This occurs when photons interact with highly doped regions within the pixel (e.g., CCD channel stops, CMOS *p* and *n*_wells and at epitaxial/substrate interface). Also backside illuminated imagers often have a finite amount of recombination loss associated with the surface accumulation layer when dealing with photons with a very short absorption length. Fortunately the lack of perfect CCE is not as detrimental to visible photon counting in that the problem only translates to a loss of spatial information.

## 4. Conclusions and Future Development

It could be tough going in lowering the “average” read noise substantially below 1 carrier rms because of the 1/*f* noise wall. CDS processing has been pushed to its limit in reducing noise from this source. Averaging multiple samples doesn’t help us since 1/*f* noise increases with time as a function of sample count increases. Using buried channel MOSFETS have been successful for reducing RTN but not for 1/*f* noise. Increasing the SN conversion gain is also restricted for the given design and process rules that must be followed. Although our SN conversion is presently limited to 160 uV/h+ there is still some wiggle room left in reducing parasitic capacitances associated with the TG and reset MOSFET that couple to the SN. However, the reduction won’t be that significant in that the SF capacitance currently dominates. Consequently, it appears the only means to lower read noise for us is to reduce 1/*f* noise head on. 

Hence, future development will focus on 1/*f* reduction without knowing where it is coming from. Various trial and error approaches will be employed on a new runs currently being fabed (be it for mobility or GR reasons). For example one lot run contains deeper buried channel MOSFETs in hopes to see a 1/*f* noise change. Special attention is being given to punch-through issues which usually emerge with buried channel use. Also in order to maximize the surface barrier potential for a buried device the SN must be reset to a SF gate bias voltage that is close as possible to substrate potential. Doing so will reduce dynamic range but the foremost objective behind these experiments is to locate the 1/*f* noise source without much regard to using the pixel in practice.

Experiments involving implants associated with the wells which contain the pixel MOSFETs are being fabricated. We can do this because the pixel implants are derived from custom reticules and independent from the standard *n* and *p*_wells used for support circuitry. As mentioned above reducing the doping concentration near the surface could lead to a more uniform current flow with less fluctuation. For example, experimental pixels being fabricated will include a single “minimum implant dose” SF MOSFET. Possibly there will be a 1/*f* noise change since this implant recipe is considerably different than the 3 implant process being used now that is intended for high speed digital MOSFETs. Also further thermal annealing is being incorporated to better activate the pixel wells. The sub carrier noise pixels in Figure 5 and Figure 6 were fabricated with a past 0.25 µm CMOS process which is known to produce lower 1/*f* noise than the 0.18 um process applied today. The 0.25 µm process used a longer thermal anneal cycle. In addition a very long and greater temperature anneal at the very start of the process is being tried to make sure the implants are fully activated. 

Native MOSFETs of various geometries are also being fabricated. The native MOSFET is known to have lower 1/*f* noise given that the SF channel doping is the epitaxial silicon [8]. Our epi silicon resistivity varies widely from 10 to 1000 ohm-cm, and hence, we should see a 1/*f* noise change. The experiment will also include baseline MOSFETs with the same geometries as the natives in order to compare 1/*f* noise levels.

Lastly we are trying a proprietary non-imaging gate oxide process offered by Jazz Semiconductor that claims to lower MOSFET 1/*f* RTN noise by 4 to 5 times compared to 0.18 um processing that is used now. All 1/*f* noise reduction approaches above will need to determine the optimum SF geometry for lowest noise performance. 

## Abbreviations

The following abbreviations are used in this manuscript:

ACC	accumulated
CCD	charge collection efficiency
CTE	charge transfer efficiency
E	photon energy (eV)
F	Fano factor
FN	Fano noise (rms carriers)
non ACC	not accumulated
PPD	pinned photo diode
PT	photo transfer
QE	quantum efficiency
RTN	random telegraph noise
S/N	signal to noise
SF	source follower
SN	sense node
TG	transfer gate
τ_D_	aCDS dominant time constant
t_s_	clamp to sample time
